# miR-508-3p concordantly silences NFKB1 and RELA to inactivate canonical NF-κB signaling in gastric carcinogenesis

**DOI:** 10.1186/s12943-016-0493-7

**Published:** 2016-01-22

**Authors:** Tingting Huang, Wei Kang, Bin Zhang, Feng Wu, Yujuan Dong, Joanna H. M. Tong, Weiqin Yang, Yuhang Zhou, Li Zhang, Alfred S. L. Cheng, Jun Yu, Ka Fai To

**Affiliations:** Department of Anatomical and Cellular Pathology, State Key Laboratory in Oncology in South China, Prince of Wales Hospital, The Chinese University of Hong Kong, Hong Kong, SAR PR China; Institute of Digestive Disease, Partner State Key Laboratory of Digestive Disease, The Chinese University of Hong Kong, Hong Kong, SAR PR China; Li Ka Shing Institute of Health Science, Sir Y.K. Pao Cancer Center, The Chinese University of Hong Kong, Hong Kong, SAR PR China; Shenzhen Research Institute, The Chinese University of Hong Kong, Shenzhen, PR China; Department of Gastroenterology, The Affiliated Drum Tower Hospital of Nanjing University, Medical School, Nanjing, PR China; School of Biomedical Sciences, The Chinese University of Hong Kong, Hong Kong, PR China; Department of Medicine and Therapeutics, The Chinese University of Hong Kong, Hong Kong, PR China

**Keywords:** NFKB1, RELA, miR-508-3p, Gastric cancer

## Abstract

**Background:**

NF-κB signaling pathway plays an important role in gastric carcinogenesis. The basic expression and functional role of NFKB1 and RELA (components of canonical NF-κB pathway) in gastric cancer (GC) have not been well elucidated. In this study, the role of NFKB1 and RELA in gastric tumorigenesis will be investigated and their regulation by microRNAs (miRNAs) will be deeply explored.

**Methods:**

The mRNA and protein expression of NFKB1 and RELA were investigated by qRT-PCR and Western blot in GC cell lines and primary tumors. The functional roles of NFKB1 and RELA in GC were demonstrated by MTT proliferation assay, monolayer colony formation, cell invasion and migration, cell cycle analysis and in vivo study through siRNA mediated knockdown. Identification of NFKB1 as a direct target of tumor suppressor miRNA miR-508-3p was achieved by expression regulation assays together with dual luciferase activity experiments.

**Results:**

NFKB1 and RELA were up-regulated in GC cell lines and primary tumors compared with normal gastric epithelium cells and their upregulation correlation with poor survival in GC. siRNA mediated knockdown of NFKB1 or RELA exhibited anti-oncogenic effect both in vitro and in vivo. NFKB1 was further revealed to be a direct target of miR-508-3p in gastric tumorigenesis and their expression showed negative correlation in primary GC samples. miR-508-3p was down-regulated in GC cells compared with normal gastric epithelium samples and its ectopic expression in GC cell lines also exerts tumor suppressor function. NFKB1 re-expression was found to partly abolish the tumor-suppressive effect of miR-508-3p in GC.

**Conclusion:**

All these findings supports that canonical NF-κB signaling pathway is activated in GC at least by the inactivation of miR-508-3p and this might have therapeutic potential in GC treatment.

**Electronic supplementary material:**

The online version of this article (doi:10.1186/s12943-016-0493-7) contains supplementary material, which is available to authorized users.

## Background

Although the incidence of gastric cancer (GC) has decreased in recent years, it is still the fourth-most-common cancer globally and the second-leading cause of the cancer deaths [1]. There are several risk factors for GC: *Helicobacter pylori* (*H. pylori*) and EBV infection, high-salt and low-vegetable diet, smoking, chronic gastritis with intestinal metaplasia [2]. According to Lauren’s classification, approximately 95 % of GC are adenocarcinomas by histological phenotype as intestinal type, diffuse type and mixed type [3]. Most GC patients are diagnosed at the advanced stage often accompanied with extensive invasion and lymphatic metastasis. Nowadays, molecular classification of GC have been proposed based on the analysis of whole-genome gene expression studies or deep sequencing studies [4]. In the development of GC, the alterations of signaling pathways are important for the tumorigenesis. Previous studies in GC revealed multiple oncogenic signaling pathways such as Wnt/β-catenin, NF-κB, Sonic Hedgehog, Notch and epidermal growth factor receptor pathway are implicated in gastric carcinogenesis [5]. To identify the novel oncogenic signaling pathway and reveal the molecule mechanisms of these pathways will facilitate to identify novel druggable targets for personalized therapy. Thus deep investigations into the signaling pathways and molecular mechanisms involving in GC progression become imperative and urgent for targeted therapy.

Mammalian NF-κB family is composed of five members, including RELA (also named p65), RELB, c-Rel, NF-κB1 p50, and NF-κB2 p52, which form various dimeric complexes that transactivates numerous target genes via binding to the κB enhancer [6]. These proteins function as dimeric transcription factors that control genes regulating a broad range of biological processes including inflammation and cancer [7–9]. The role of NF-κB activation in tumor progression, cell growth, and apoptosis may differ according to species and cell type [10]. NF-κB is reported to play an important role in the induction of cytokine expression and promote progression of GC [11], and its activation correlates with chronic inflammation and tumorigenesis induced by *H. pylori* for gastric tumor [12, 13]. Furthermore, by using transgenic mice possessing an NF-κB-responsive lacZ reporter gene, the responses of mouse host cells to *H. pylori* infection were investigated in vivo. It was suggested that *H. pylori* may be able to regulate NF-κB signaling during chronic infection [14]. However the reports on clinical significance of NF-κB in GC seem controversial. Some groups demonstrated activated NF-κB correlates with better prognosis in early-stage GC [15], whereas some groups reported the NF-κB upregulation and nuclear accumulation correlates with poor survival. In our preliminary study, we found that NFKB1 and RELA protein (key components of canonical NF-κB pathway) levels were upregulated but there are no significant differences between normal control and cancerous tissues from mRNA expression, suggesting the translational or post-translational regulation play important role for the upregulated protein expression of NF-κB.

microRNAs (miRNAs) are a kind of small non-coding RNAs which have been identified as new regulators of gene expression through binding to the 3' untranslated regions (UTRs) of the target mRNA [16]. This results in mRNA degradation or translational inhibition. Emerging evidence have showing that miRNAs are abnormally expressed in various cancers [17], and the deregulated miRNA expressions are strongly associated with tumor initiation, promotion and progression [18, 19]. The protein upregulation of NFKB1 but not from mRNA level suggested miRNA might play a role in the regulation of NFKB1 in GC. By TargetScan (www.targetscan.org) miR-508-3p are found to have several putative targets including NFKB1 which has a binding site of miR-508-3p in its 3'UTR (8mer, total context + score −0.34). And this was also predicted by miRDB (http://mirdb.org/miRDB/) with a target score 75. Thus we proposed that NFKB1 might be negatively regulated by miR-508-3p.

In current study, we will first investigate the basic expression patterns and functional roles of NFKB1 and RELA in GC. Furthermore, miR-508-3p will be identified as a negative regulator of NF-κB pathway by targeting NFKB1. All our findings were proposed to provide the first evidence that canonical NF-κB pathway is activated in GC at least due to the downregulation of miR-508-3p and this might have clinical intervention potential.

## Results

### NFKB1 and RELA are up-regulated in GC cell lines and primary tumors

**Fig. 1:**
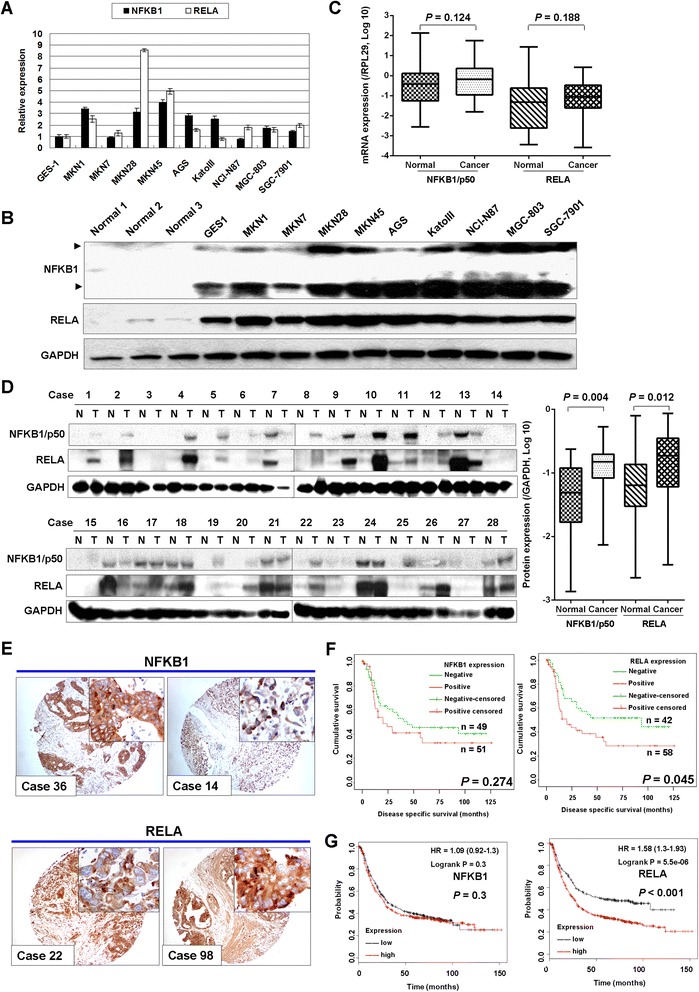
NFKB1 and RELA are up-regulated in GC cell lines and primary gastric tumors. **a** The mRNA expression of NFKB1 and RELA in nine GC cell lines compared with GES-1 cells (immortalized gastric epithelium cell line). The standard deviations (SDs) were achieved by qRT-PCR (−Delta Delta Ct values) in triplicate wells. **b** Western blot analysis of NFKB1 and RELA in nine GC cell lines, GES-1 cells and three normal gastric tissues (Normal 1–3 protein samples are from normal gastric mucosa obtained from weight reduction gastric surgery). **c** NFKB1 and RELA mRNA expression in 28 paired primary GC samples (NFKB1, *P* = 0.124; RELA, *P* = 0.188). **d** NFKB1 and RELA showed increased protein expression in primary gastric tumors compared with paired non-tumours adjacent tissues (*n* = 28; NFKB1, *P* = 0.004; RELA, *P* = 0.012). **e** Representative immunohistochemistry images of positive NFKB1 and RELA expression in GC tissue microarray (original magnification × 100, insertion × 400). NFKB1 and RELA expression are mainly localized in the cytoplasm. **f** Kaplan-Meier plots of disease specific survival according to NFKB1 or RELA expression status. RELA accumulation in cytoplasm was associated with poor disease specific survival in patients with GC (*P* = 0.045), but NFKB1 overexpression only correlated with a non-significant trend of poor survival (*P* = 0.274). **g** The prognosis plots according to NFKB1 and RELA mRNA expression in GC (from KM plotter). Only high RELA mRNA expression significantly correlated with overall survival in 876 GC samples (*P* < 0.001)

The expression of NFKB1 and RELA were detected in nine GC cell lines as well as normal gastric epithelial samples by qRT-PCR and Western blot analysis. Both NFKB1 and RELA mRNA expression were observed up-regulated in most GC cell lines with qRT-PCR compared with immortalized gastric epithelium cell line (GES-1) (Fig. 1a). The up-regulation of NFKB1 and RELA protein expression were also detected in all nine GC cell lines by Western blot analysis. In contrast, the 3 non-neoplastic gastric tissue samples showed weak expression of NFKB1 and RELA protein (Fig. 1b). In 28 paired clinical GC samples, the NFKB1 and RELA mRNA expression level had no significant difference in tumor VS normal (*P* = 0.124 and *P* = 0.188 respectively) (Fig. 1c). However, both NFKB1 and RELA protein expression showed up-regulated in tumor samples compared with paired normal gastric tissues after quantified normalization (*P* = 0.004 and *P* = 0.012 respectively) (Fig. 1d).

Immunohistochemistry was performed to assess the NFKB1 and RELA protein expression in 100 primary GC samples in tissue-microarray. Both NFKB1 and RELA were mainly localized in the cytoplasm of the tumor cells (Fig. 1e). Positive immunoreactivity was observed in 51 (51 %) and 58 (58 %) gastric adenocarcinomas for NFKB1 and RELA staining respectively. Expression of RELA in GC was associated with poorer disease specific survival by univariate analysis (*P* = 0.045, Fig. 1f), but upregulation of NFKB1 only correlated with a non-significant trend of poor prognosis (*P* = 0.274). The clinicopathologic characteristics of 100 patients with GC and the association with NFKB1 and RELA expression were shown in Table 1. NFKB1-positive tumors were more likely to be found in elder age group (*P* = 0.052), but RELA-positive tumor are strongly correlated with N stage (*P* = 0.042). Univariate analysis indicated that old age (*P* = 0.041), histology with diffuse component (*P* < 0.000), grade (*P* = .0018), T stage (*P* < 0.000), N stage (*P* < 0.000), M stage (*P* < 0.000) correlated with poor disease specific survival. By multivariate Cox proportional hazards regression analysis, only T stage, N stage and M stage were independently associated with disease specific survival (Table 2).

**Table 1:** Correlation of NFKB1 and RELA expression with clinicopathologic parameters (significant *P-*value in bold and Italic format; *#*, marginally significant)

		NFKB1 expression			RELA expression		
		Negative number (%)	Positive number (%)	*P*-value	Negative number (%)	Positive number (%)	*P*-value
Sex	M	30 (44.1)	38 (55.9)	0.199	25 (36.8)	43 (63.2)	0.135
	F	19 (59.4)	13 (40.6)		17 (53.1)	15 (46.9)	
Age	<=60	20 (64.5)	11 (35.5)	***0.052#***	15 (48.4)	16 (51.6)	0.392
	>60	29 (42.0)	40 (58.0)		27 (39.1)	42 (60.9)	
Type	Intestinal	32 (49.2)	33 (50.8)	1.000	26 (40.0)	39 (60.0)	0.672
	Diffuse	17 (63.0)	10 (37.0)		16 (45.7)	19 (54.3)	
Grade	1	2 (40.0)	3 (60.0)	0.475	1(20.0)	4 (80.0)	0.252
	2	18 (42.9)	24 (57.1)		15 (35.7)	27 (64.3)	
	3	29 (54.7)	24 (45.3)		26 (49.1)	27 (40.9)	
Stage (T)	1	3 (50.0)	3 (50.0)	0.884	4 (66.7)	2 (33.3)	0.456
	2	14 (48.3)	15 (51.7)		13 (44.8)	16 (55.2)	
	3	27 (47.4)	30 (52.6)		23 (40.4)	34 (59.6)	
	4	5 (55.6)	3 (44.4)		2 (25.0)	6 (75.0)	
Stage (N)	0	10 (45.5)	12(54.5)	0.605	12 (54.5)	10 (45.5)	***0.042***
	1	13 (61.9)	8 (38.1)		12 (57.1)	9 (42.9)	
	2	15 (44.1)	19 (55.9)		8 (23.5)	26 (76.5)	
	3	11 (47.8)	12 (52.2)		10 (43.5)	13 (66.5)	
Stage (M)	0	40 (48.2)	43 (51.8)	0.794	34 (41.0)	49 (59.0)	0.788
	1	9 (52.9)	8 (47.1)		8 (47.1)	9 (52.9)	
Lymph Node	0	10 (45.5)	12 (54.5)	0.811	12 (54.5)	10 (45.5)	0.223
	1	39 (50.0)	39 (50.0)		30 (38.5)	48 (61.5)	
*H. pylori*	Absence	19 (54.3)	16 (45.7)	0.530	14 (40.0)	21 (60.0)	0.834
	Presence	30 (46.2)	35 (53.8)		28 (43.1)	37 (56.9)

**Table 2:** Univariate and multivariate Cox regression analysis of clinicopathologic factors in 100 patients with GC (significant *P*-value in bold and Italic format)

	Univariate	Multivariate
Sex	0.285	
Age	***0.041***	0.218
Type	***<0.000***	0.366
Grade	***0.018***	0.527
Stage (T)	***<0.000***	***0.004***
Stage (N)	***<0.000***	***<0.000***
Stage (M)	***<0.000***	***0.005***
*H. pylori*	0.210	
NFKB1	0.274	
RELA	***0.045***	0.155

The prognosis significance of NFKB1 and RELA mRNA expression in GC was achieved by KM plotter (http://kmplot.com/analysis/) [20]. In 876 GC patients, the high expression of NFKB1 was not significantly correlated with poor overall survival, but still had a prognostic trend (*P* = 0.3, HR = 1.09). Meanwhile, RELA upregulation was strongly associated with unfavorable outcome as a prognostic marker (*P* < 0.001, HR = 1.58, Fig. 1g).

### NFKB1 and RELA knockdown in GC exert tumor suppressor effect both in vitro and in vivo

**Fig. 2:**
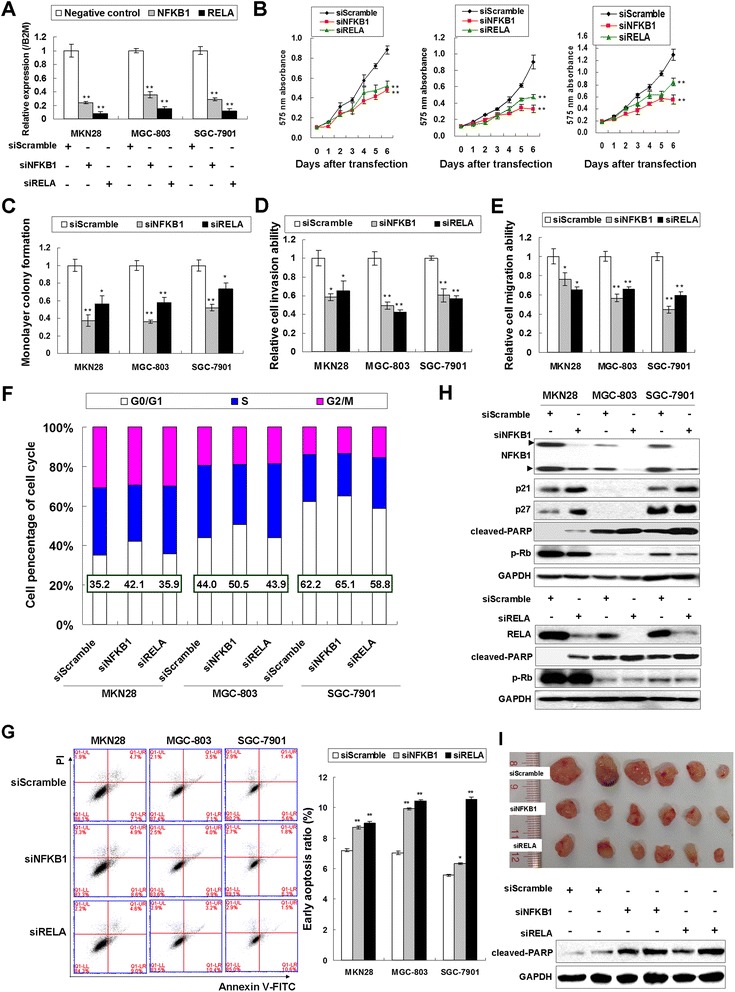
NFKB1 and RELA knockdown exerts tumor suppressor function in GC cells. **a** Transfection with siNFKB1 or siRELA decreased the mRNA expression of NFKB1 and RELA respectively in MKN28, MGC-803 and SGC-7901 cells. **b** 6-day MTT assays revealed NFKB1 or RELA knockdown significantly suppressed proliferation rate of GC cells (**, *P* < 0.001). **c** siNFKB1 or siRELA decreased monolayer colony formation in MKN28, MGC-803 and SGC-7901 cells (*, *P* < 0.05; **, *P* < 0.001). The experiments were performed in triplicate wells to get SDs. **d** NFKB1 or RELA knockdown inhibited GC cell invasion (*, *P* < 0.05; **, *P* < 0.001). Three random vision fields were selected for invaded cell counting to get SDs. **e** The GC cell migration abilities were suppressed by siNFKB1 or siRELA (*, *P* < 0.05; **, *P* < 0.001). The cells were counted in three random vision fields to get SDs. **f** Flow cytometry analysis of NFKB1 or RELA knockdown transfectants together with scramble siRNA transfectants as control. Two independent experiments were performed and the representative one was shown in the bar chart. **g** Effects of siNFKB1 and siRELA on the induction of early apoptosis (Annexin V-FITC and PI double-staining). The cell population with early apoptosis was shown in the lower right of each treatment. siNFKB1 or siRELA induced early apoptosis compared with siScramble control after 20-h transfection (*, *P* < 0.05, **, *P* < 0.001). **h** *Upper*, Western blot analysis of NFKB1, p21, p27, cleaved-PRAP and p-Rb after siNFKB1 transfection; *Lower*, the Western blot result of RELA, cleaved-PRAP and p-Rb in the transfectants of siRELA. **i** siNFKB1 and siRELA formed smaller xenografts than siScramble using MGC-803 cells in a 28-day inoculation and the elevated cleaved-PARP was detected in siNFKB1 and siRELA xenografts

To investigate the functional role of NFKB1 and RELA in GC cells, siRNA-mediated knockdown in MKN28, MGC-803 and SGC-7901 cells was performed. A significantly decreased NFKB1 and RELA mRNA expression was observed in these cell lines transfected with siNFKB1 or siRELA when compared with scramble siRNA groups (Fig. 2a). Both NFKB1 and RELA knockdown suppressed MKN28, MGC-803 and SGC-7901 cell proliferation in a 6-day MTT assay (Fig. 2b). Monolayer colony formation assay indicated that NFKB1 and RELA knockdown significantly reduced colony formation in these three cell lines (Fig. 2c). Moreover, siNFKB1 and siRELA inhibited both cell invasion and migration of GC cells (Fig. 2d and e, Additional file 1: Figure S1A and S1B). Since a growth inhibitory effect was observed in siNFKB1 and siRELA-transfected cells, the transfectants for cell-cycle parameters were analyzed by flow cytometry. Twenty-four hours after transfection, accumulation of G0/G1 cells increased in siNFKB1 transfectants compared with the scramble siRNA controls. As shown in Fig. 2f, 42.1 % of siNFKB1 treated MKN28 cells were in G1 phase but only 35.2 % of scramble siRNA control cells were in G1 phase after transfection. As the same, SGC-803 and SGC-7901 cells with NFKB1 knockdown contained a higher percentages of G1 phase cells (50.5 % and 65.1 %) compared with siScramble control counterparts (44 % and 62.2 % respectively). However, the cell population of G1 phase in RELA-knockdown MKN28, MGC-803 and SGC-7901 cells were 35.9, 43.9 and 58.8 % respectively. Therefore in siRELA transfectants, there were no obvious changes for the ratio of G0/G1 phase (Fig. 2f and Additional file 1: Figure S1C).

Annexin V-FITC and Propidium iodide [21] double-staining were performed for early apoptosis analysis after siNFKB1 or siRELA treatment in MKN28, MGC-803 and SGC-7901 cells. As shown in Fig. 2g, cells in the lower right quadrant indicated Annexin V-positive/PI-negative early apoptotic cells. The percentage of early apoptosis cells shows increased trends in all the three GC cell lines upon siNFKB1 and siRELA treatment. The population of early apoptosis cells in siNFKB1 transfectants increased to 8.6, 9.9 and 6.3 % in three cell lines respectively compared with siScramble control cells (7.7, 7.1 and 5.6 % respectively in MKN28, MGC-803 and SGC-7901 cells). RELA knockdown also induced early apoptosis in these three GC cell lines to 9.0, 10.4 and 10.8 % respectively.

The associated cell cycle regulators were also analyzed by Western blot. It has been shown that Rb protein is responsible for a major G1 checkpoint, promoting S-phase entry and cell growth. The phosphorylated Rb (p-Rb) expression was decreased but p21 and p27 were uniformly up-regulated in NFKB1 knockdown cells, supporting the G0/G1-phase cell cycle arrest determined by cell cycle analysis. However, p-Rb activation showed no obvious change after RELA knockdown. Moreover, both siNFKB1 and siRELA induced late apoptosis, represented by the activation of cleaved-PARP in all the three GC cell lines MKN28, MGC-803 and SGC-7901 (Fig. 2h).

The effect of NFKB1 and RELA expression on in vivo tumor growth was also studied. MGC-803 cells transfected with scramble siRNA, siNFKB1 or siRELA were subcutaneously injected into four-week-old male nude mice. The tumor formation was monitored and documented every 6 days. Tumors grew slower and showed smaller size in siNFKB1 or siRELA group than those in the scramble siRNA group after 28-day inoculation (Fig. 2i and Additional file 1: Figure S1D). In addition, the elevated cleave-PARP was detected in siNFKB1 and siRELA group compared with siScramble group, suggesting NFKB1 and RELA knockdown also induced late apoptosis in vivo.

### NFKB1 is a direct target of miR-508-3p in GC

**Fig. 3:**
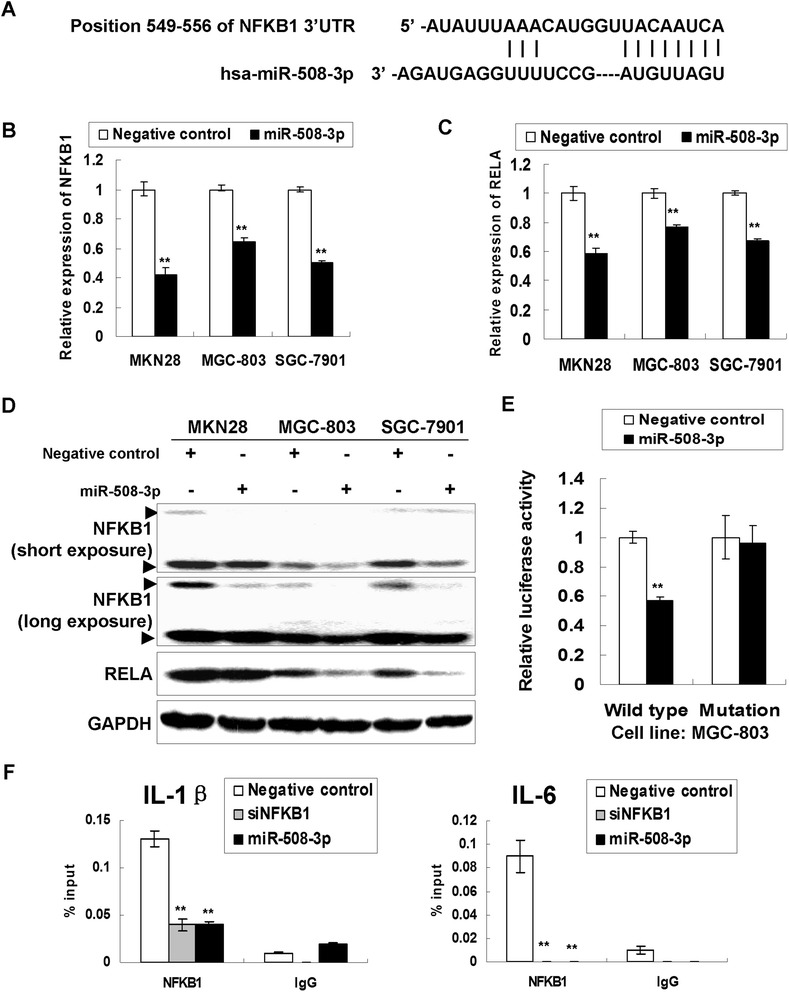
NFKB1 is a direct target of miR-508-3p in GC. **a** The binding site in the NFKB1 3'UTR for miR-508-3p as predicted by TargetScan (www.targetscan.org). **b** NFKB1 mRNA expression was down-regulated by ectopic miR-508-3p expression in MKN28, MGC-803 and SGC-7901 cells (**, *P* < 0.001). **c** Ectopic miR-508-3p expression decreased the RELA mRNA expression in GC cells (**, *P* < 0.001). **d** Both NFKB1 and RELA protein were down-regulated by miR-508-3p in three GC cell lines. **e** miR-508-3p overexpression inhibited the luciferase activity in the constructs containing wild type binding site, but the luciferase activity in the construct containing mutated binding site of NFKB1 3'UTR was not affected (Wild type, the construct containing the complementary sequence of seed region; Mutation, the binding site was deleted; **, *P* < 0.001). **f** ChIP-qPCR analysis on the promoter region of IL-1β and IL-6 after treating the cells with siNFKB1 or miR-508-3p. siNFKB1 or miR-508-3p decreased the binding affinity of NFKB1 on the promoter region of downstream targets IL-1β and IL-6 (**, *P* < 0.001). IP by IgG was as experimental negative control

miR-508-3p was found to have multiple putative targets including NFKB1 which has a binding site for miR-508-3p in its 3'UTR. The putative binding site of miR-508-3p with NFKB1 3'UTR was shown in Fig. 3a. The mRNA expression of NFKB1 and RELA was found decreased in MKN28, MGC-803 and SGC-7901 cells after ectopic expression of miR-508-3p (Fig. 3b and c). NFKB1 and RELA protein also showed a decrease expression after miR-508-3p overexpression, indicating that miR-508-3p triggered a silencing effect on the endogenous NFKB1 and RELA both from mRNA and protein level (Fig. 3d).

To test whether NFKB1 was a direct target of miR-508-3p, the 3'UTR binding site fragments of NFKB1 were directly fused to the downstream of the firefly luciferase gene of pMIR-REPORT vector. As shown in Fig. 3e, miR-508-3p inhibited the relative luciferase activity of construct encompassing NFKB1 3'UTR binding site, but it had no effect on the construct containing mutated sequence of the binding site. This results supported that miR-508-3p might recognize the binding sites in NFKB1 3'UTR and directly suppressed NFKB1 expression.

To further investigate if miR-508-3p or siNFKB1 also regulates the binding affinity of NFKB1 on the promoter region of downstream targets (IL-1β and IL-6), ChIP-qPCR was performed in SGC-7901 cells. Equal DNA fragments were loaded and the qPCR revealed the binding affinity of NFKB1 with IL-1β/IL-6 was significantly decreased (Fig. 3f), suggesting miR-508-3p inhibits the DNA binding ability of NFKB1 by down-regulating NFKB1.

### miR-508-3p is down-regulated and functions as potential tumor suppressor in GC cells

**Fig. 4:**
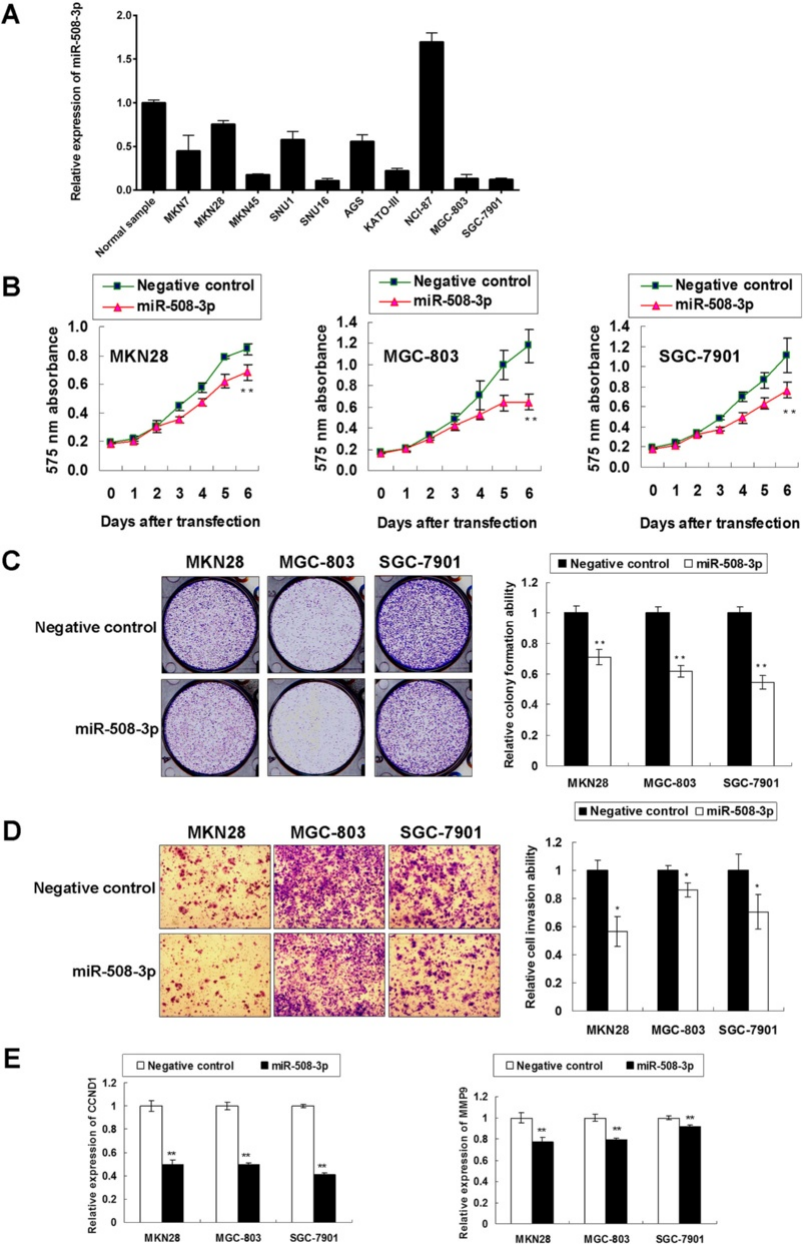
miR-508-3p is down-regulated in GC cell lines and has tumor suppressor potential. **a** The expression of miR-508-3p in ten GC cell lines compared with commercial normal gastric total RNA (AM7996, Ambion). **b** 6-day MTT proliferation assays revealed the growth-inhibition effect of miR-508-3p in MKN28, MGC-803 and SGC-7901 cells (**, *P* < 0.001). **c** Ectopic miR-508-3p expression decreased monolayer colony formation in all the three GC cell lines (**, *P* < 0.001). The experiments were performed in three wells to get SDs. **d** The GC cell invasion ability was significantly inhibited by ectopic expression of miR-508-3p (*, *P* < 0.05). The invaded cells from the matrigel were counted in three random vision fields for getting SDs. **e** The mRNA expression of CCND1 and MMP9 was down-regulated upon ectopic miR-508-3p expression in MKN28, MGC-803, and SGC-7901 cells (**, *P* <0.001)

The expression level of miR-508-3p was examined in ten GC cell lines. miR-508-3p showed decreased in nine GC cell lines compared with normal gastric epithelium sample (Fig. 4a). Downregulation of miR-508-3p expression in GC indicated it might have tumor suppressor function in gastric carcinogenesis and the functional studies were performed by ectopic expression of miR-508-3p precursor. miR-508-3p significantly suppressed cell growth of all the three GC cell lines in a 6-day MTT assay (Fig. 4b). The growth suppressive function of miR-508-3p was further validated by monolayer colony formation assay. A reduction of colony number was observed in miR-508-3p transfectants compared with scramble miRNA groups (Fig. 4c).

miR-508-3p also significantly inhibited cell invasion ability of MKN28, MGC-803 and SGC-7901 cells. The GC cells were ectopic expressed with miR-508-3p precursor and the cell invasion was measured. miR-508-3p overexpression resulted in a decreased invasion ability to 56.7, 86.3 and 70.5 % compared to negative control group (Fig. 4d). The decreased CCND1 mRNA was observed in miR-508-3p transfected MKN28, MGC-803 and SGC-7901 cells, elucidating the proliferation-inhibition effect of miR-508-3p in gastric tumorigenesis. Furthermore, miR-508-3p dramatically inhibited the mRNA expression of MMP9, which functions as an enhancer for cell invasion by promoting the degradation of extracellular matrix (Fig. 4e).

### NFKB1 re-expression partly counteracts the tumor-suppressive effect of miR-508-3p

**Fig. 5:**
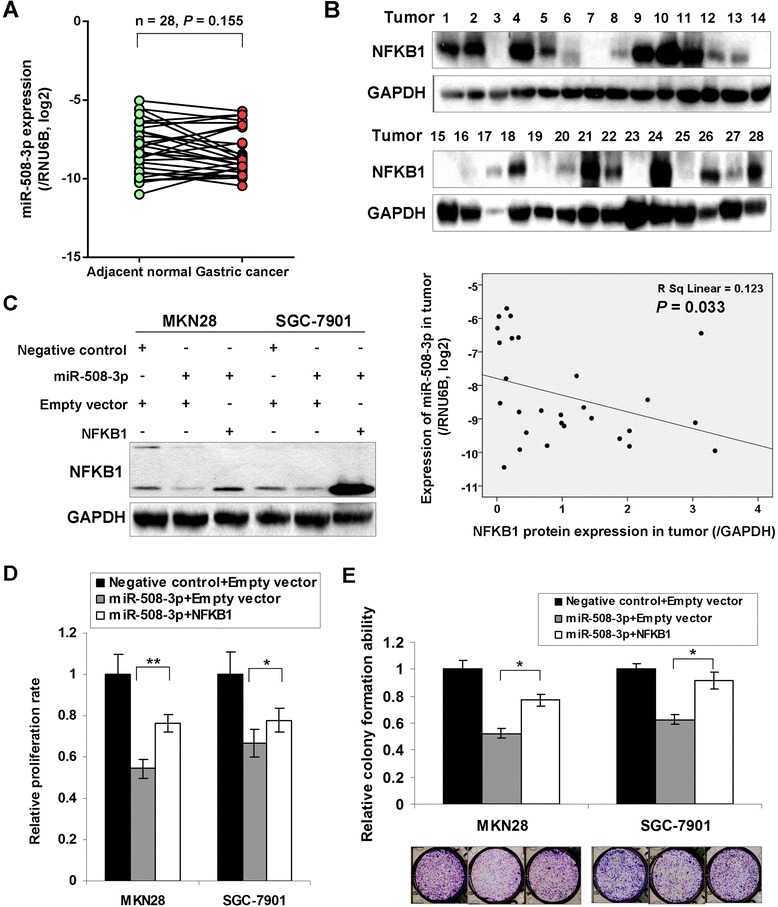
NFKB1 re-expression partly abrogated the inhibitory effect of miR-508-3p in GC. **a** Expression of miR-508-3p in paired primary GC samples (*n* = 28; *P* = 0.155). **b** miR-508-3p expression was negatively correlated with NFKB1 protein expression in primary gastric tumors (*P* = 0.033). **c** The Western blot analysis of NFKB1 in the rescue experiments. **d** NFKB1 re-expression promoted cell proliferation compared with miR-508-3p alone in MKN28 and SGC-7901 cells (*, *P* < 0.05; **, *P* < 0.001). The SDs were get by the 575 nm absorbance readings in 4 wells of each item. **e** NFKB1 re-expression counteracted proliferation-inhibition effect of miR-508-3p revealed by monolayer colony formation assays revealed (*, *P* < 0.05). The representative colony formation figures were shown in the bottom

In 28 paired primary GC samples, miR-508-3p showed a non-significant trend of downregulation in adenocarcinoma compared with corresponding adjacent non-tumorous mucosae (*P* = 0.155; Fig. 5a). As miR-508-3p showed decreased expression whereas NFKB1 showed up-regulated expression in GC, the expression correlation of miR-508-3p and NFKB1 was analyzed in 28 paired fresh samples. NFKB1 protein expression shows negatively correlation with miR-508-3p in tumor tissues by Pearson correlation analysis (*P* = 0.033, Fig. 5b). This result suggested that the downregulation of miR-508-3p was partly responsible for endogenous NFKB1 overexpression in GC. NFKB1 re-expression in rescuing the suppressive phenotypes of GC cells by ectopic expression of miR-508-3p was further investigated (Fig. 5c). Interestingly, the growth inhibitory phenotypes were partly counteracted by NFKB1 re-expression in MKN28 and SGC-7901 cells (MTT proliferation assay, *P* < 0.05, Fig. 5d; monolayer colony formation assay, *P* < 0.05, Fig. 5e).

## Discussion

NF-κB signaling pathway has been reported to be activated in GC due to *H. pylori* infection [22]. *H. pylori* promotes degradation of IκBα, a cytoplasmic inhibitor of NF-κB. In kinase assay, *H. pylori* induced IKKα and IKKβ catalytic activity in GC cells thus to activate NF-κB pathway [22, 23]. *H. pylori* infection also enhances gastric epithelial cells invasion by activating MMP9 and VEGF expression, which was mediated through a NF-κB and COX-2 mediated pathway [24].

NF-κB activation is strongly correlated with enhanced cell invasion/migration and anti-apoptosis [25] and NF-κB is proposed in the centre of functions exerted by oncogenes or tumor suppressor genes. TGF-α enhanced the expression of anti-apoptotic Bcl-2 family proteins in an NF-κB dependent manner [26]. Connective tissue growth factor (CTGF) [27], interleukin 17A (IL-17A) [28], miR-362 [29] and high mobility group box 1 (HMGB1) [30] promote GC invasion and metastasis through modulating the NF-κB pathway. Loss of tumor suppressor gene TFF1 leads to activation of IKK complex-regulated NF-κB transcription factors and is an important event in shaping the NF-κB-mediated inflammatory response during the progression to gastric tumorigenesis [21]. Other tumor suppressor genes, inhibitor of growth 4 (ING4) [31], Metallothionein 2A (MT2A) [32], Sirtuin 1 (SIRT1) [33, 34], FOXP3 [35] and Gastrokine 1 (GKN1) [36] also exerts their proliferation and invasion inhibition function though suppression of NF-κB signaling pathway.

Although NF-κB was confirmed to play an important role in gastric tumorigenesis, no comprehensive study was performed to reveal the expression pattern of canonical and non-canonical NF-κB in GC. In this study, we found the components of canonical NF-κB signaling pathway, NFKB1 and RELA, are strongly up-regulated from the protein but not from mRNA level in GC samples, suggesting miRNA regulation might play an important role in the regulation of NF-κB pathway. Meanwhile, the prognostic significance analysis revealed the RELA upregulation was associated with poor survival in GC, which was concordant with the previous studies [12, 37]. From the functional study by siRNA-mediated knockdown, we comprehensively revealed the functional role of NFKB1 and RELA in GC. Functional studies demonstrated that downregulation of NFKB1 and RELA expression by siRNA quenched their oncogenic properties by inhibiting cell growth in vitro, inducing G1 phase accumulation (only siNFKB1) and apoptosis. Furthermore, NFKB1 and RELA knockdown inhibited cell invasion and migration and suppressed xenograft formation in vivo. miR-508-3p, which is listed in the top rank of putative regulators of NFKB1 from several bioinformatic websites, was first identified to be a negative regulator of NF-κB pathway through direct targeting NFKB1.

miR-508-3p (member of the miR-506 family) is located on Xq27.3, which is a fragile site of the human X chromosome. The function of miR-508-3p is not well elucidated. The very limited reports about miR-508-3p are controversial according to different cancer types. In renal cell carcinoma (RCC), the level of miR-508-3p demonstrated significant decreased expression [38]. Ectopic expression of miR-508-3p suppressed the proliferation of RCC cells, induced cell apoptosis and inhibited cell migration in vitro. In esophageal squamous cell carcinoma (ESCC), the elevated miR-508-3p correlates with poor survival and activated PI3K/Akt signaling by targeting inositol polyphosphate-5-phosphatase J (INPP5J), phosphatase and tensin homologue (PTEN) and inositol polyphosphate 4-phosphatase type I (INPP4A) [39]. In this study, it was first discovered that miR-508-3p was down-regulated across a panel of GC cell lines and primary tumors compared with normal gastric epithelium, which suggested its tumor suppressor potential roles in gastric tumorigenesis. Functional study demonstrated ectopic expression of miR-508-3p suppressed GC cell proliferation, reduced monolayer colony formation and inhibited cell invasion. In addition, the expression of miR-508-3p showed negative correlation with NFKB1 protein expression in tumor tissues and NFKB1 re-expression partly abolished the inhibitory effect of miR-508-3p in GC. All these findings confirmed the critical tumor suppressor role of miR-508-3p by targeting NF-κB pathway in gastric carcinogenesis.

## Conclusions

In conclusion, we first identified that miR-508-3p downregulation contributes to canonical NF-κB activation in gastric tumorigenesis. The findings of this study not only enhance our understanding of the underlying mechanism of GC development, but also may potentially lead to the development of useful tumor markers for GC and specific intervention strategies based on the recognized regulatory pathways.

## Methods

### Cell lines and primary gastric tissues

Human GC cell lines (MKN1, MKN7, MKN28, MKN45, AGS, KatoIII, NCI-N87, MGC-803, SGC-7901) and one immortalized gastric epithelial cell line (GES-1) have been described in previous study [40]. Cells were cultured at 37 °C in humidified air atmosphere containing 5 % CO_2_ in RPMI 1640 (GIBCO, Grand Island, NY) medium supplemented with 10 % fetal bovine serum (GIBCO). The 28 primary paired samples (tumor samples and adjacent non-tumorous samples) from GC patients were randomly chosen from Prince of Wales Hospital (Year 2009–2010). Ethical approval was obtained from the Joint Chinese University of Hong Kong-New Territories East Cluster Clinical Research Ethics Committee (CREC Ref. No: 2015.269).

### Protein extraction and Western blot analysis

Protein was extracted from GC cell lines and paired primary tissues using RIPA lysis buffer with proteinase inhibitor. Protein concentration was measured by the method of Bradford (Bio-Rad, Hercules, CA) and 20 μg of protein mixed with 2 × SDS loading buffer was loaded per lane, separated by 12 % SDS-polyacrylamide gel electrophoresis. The primary antibodies used in this study includes NFKB1 (#3035, Cell Signaling), RELA (#3034, Cell Signaling), p21 (#2946, Cell Signaling), p27 (#2552, Cell Signaling), p-Rb (Ser807/811) (#9308, Cell Signaling), cleaved-PARP (Asp214) (#9541, Cell Signaling) and GAPDH (#2118, Cell Signaling). The secondary antibodies were anti-Mouse IgG-HRP (00049039, Dako, 1:30000) and anti-Rabbit IgG-HRP (00028856, Dako, 1:10000). The Western blot bands were quantified by ImageJ.

### RNA extraction and quantitative real-time polymerase chain reaction (qRT-PCR)

Total RNA from tissue samples and cultured cells was extracted using TRIzol reagent (Invitrogen, Carlsbad, CA). High-Capacity cDNA Reverse Transcription Kits (Applied Biosystems, Carlsbad, CA) were used for cDNA synthesis. qRT-PCR was used to quantitative differences in mRNA expression of associated genes and primers were listed as following: NFKB1 (sense: GGC AGC ACT ACT TCT TGA CC; anti-sense: CAG CAA ACA TGG CAG GCT AT); RELA (sense: GCC TGT CCT TTC TCA TCC CA; anti-sense: CTG CCA GAG TTT CGG TTC AC); CCND1 (sense: CCC TCG GTG TCC TAC TTC AA; anti-sense: CTC CTC GCA CTT CTG TTC CT); MMP9 (sense: GCA GTA CCA CGG CCA ACT A; anti-sense: GCC TTG GAA GAT GAA TGG AA); B2M (sense: ACT CTC TCT TTC TGG CCT GG; anti-sense: ATG TCG GAT GGA TGA AAC CC). The relative expression level was normalized and calculated by B2M using the 2^ (−Delta Delta Ct) method. PCR was performed using SYBR Green PCR reagents (Applied Biosystems) according to the manufacturer’s instructions. The reactions were incubated in a 96-well plate at 95 °C for 10 min, followed by 40 cycles of 95 °C for 15 s and 60 °C for 1 min.

For microRNA expression detection, Taqman miRNA assays were used to quantify the expression levels of mature miR-508-3p (Assay ID: #001052, Life Technologies). The relative expression level of microRNAs was normalized by RNU6B (Assay ID: #001093, Life Technologies). The reactions were performed in 7500 Fast Real-Time System (Applied Biosystems) and the reaction mix was incubated at 95 °C for 30 s, followed by 40 cycles of 95 °C for 8 s and 60 °C for 30 s.

### miRNA/siRNA (small inference RNA) transfection and functional study

The miRNA precursors, miR-508-3p (PM11033), scramble control (AM17110) were purchased from Life Technologies. siNFKB1 (SI02654932) and siRELA (SI0301672) were obtained from Qiagen (Valencia, CA). All transfection assays were performed using Lipofectamine 2000 Transfection Reagent (Invitrogen). Cell proliferation was assessed using CellTiter 96 Non-Radioactive Cell Proliferation Assay (Promega, Madison, WI) according to manufacturer’s instruction. For colony formation assays in monolayer cultures, the transfected cells were cultured in 6-well plates for 10 days. Cells were fixed with 70 % ethanol for 15 min and stained with 2 % crystal violet. Colonies with more than 50 cells per colony were counted. The experiments were repeated in triplicate wells to get standard deviations. The cell invasion assays using BD Biocoat Matrigel Invasion Chambers (BD Biosciences, Franklin Lakes, NJ) has been described previously by W. Kang [41]. Cell cycle analysis was performed using flow cytometry as described previously [42]. For the early apoptosis detection by flow cytometry, the cells were treated with siNFKB1, siRELA or siScramble for 20 h before sorting with Annexin-V FITC and PI double-staining.

### In vivo tumorigenicity model

The tumor-forming MGC-803 cells (10^7^ cells suspended in 100 μl PBS) transiently transfected with scramble control or siNFKB1 and siRELA were injected subcutaneously into dorsal flank of 4-week old Balb/c nude mice respectively. When the tumors were palpable in Day 4, the synthetic siRNA complex (25 nM) with siPORT Amine transfection reagent (Ambion) in 30 μl PBS was delivered intratumorally in 6-day-interval. Tumor diameter was measured and documented every 6 days until the end of Day 28. The xenografts were collected for Western blot analysis of cleaved-PARP. Tumor volume (mm^3^) was estimated by measuring the longest and shortest diameter of the tumor and calculating as follows: volume = (shortest diameter)^2^ × (longest diameter) × 0.5. All animal handling and experimental procedures were approved by Department of Health, Hong Kong (Reference No: 15–229 in DH/HA&P/8/2/1 Pt.48) and the Animal Ethics Committee of the CUHK (Reference No: 15-127-DRG).

### Immunohistochemistry

Immunohistochemistry was performed using 4 μm-thick sections of tissue microarray. After de-waxing in xylene and graded ethanol, sections were subsequently undergone microwaving in EDTA antigen retrieval buffer. The immunohistochemistry (1:100 for the primary antibodies described in Western blot analysis part) was conducted in Ventana Nex ES automated Stainer (Ventana Corporation). The cytoplasmic expression of NFKB1 and RELA was assessed by assigning a proportion score and an intensity score. The proportion score was according to proportion of tumor cells with positive cytoplasmic staining (0, none; 1, <=10 %; 2, 10 to < =25 %; 3, >25 to 50 %; 4, >50 %). The intensity score was assigned for the average intensity of positive tumor cells (0, none; 1, weak; 2, intermediate; 3, strong). The cytoplasmic score of NFKB1 and RELA was the product of proportion and intensity scores, ranging from 0 to 12. The cytoplasmic staining was categorized into negative (score 0–4) and positive (score 6–12).

### Luciferase activity assays

The putative miR-508-3p binding site at the 3'UTR of NFKB1 was subcloned into pMIR-REPORT Vector (Ambion). The oligonucleotides that encompasses the miR-508-3p recognition site are as following (sense: CTA GTA CTT GTC AAT ATT TAA ACA TGG TTA CAA TCA TTG CTG AAA GAG CT; anti-sense: CTT TCA GCA ATG ATT GTA ACC ATG TTT AAA TAT TGA CAA GTA). The oligonucleotides which contain the mutated binding site are as following (sense: CTA GTA CTT GTC AAT ATT TAA ACA TGG TTT GCT GAA AAT GGA GCT; anti-sense: CCA TTT TCA GCA AAC CAT GTT TAA ATA TTG ACA AGT A). The oligonucleotides were annealed in 30 mmol/L HEPES buffer containing 100 nmol/L potassium acetate and 2 mmol/L magnesium acetate. The firefly luciferase construct was co-transfected with Renilla luciferase vector control into MGC-803 cells. Dual luciferase reporter assays (Promega, Madison, WI) were performed 36 h after transfection.

#### ChIP-qPCR

ChIP-qPCR (chromatin immunoprecipitation followed by qPCR) was performed as described previously [43]. Briefly, SGC-7901 cells (transfected with Negative control, siNFKB1 and miR-508-3p respectively) were fixed in 1.5 % Final formaldehyde/PBS for 10 min at room temperature and quenched by glycine. After cell lysis, the chromatin was fragmented into 100–500 bp by Bioruptor Sonicator (Diagenode) and protein-DNA complexes were immunoprecipitated by 5 μg NFKB1 antibody or 2 μg anti-IgG antibody (Cell Signaling) conjugated with Dynalbeads Protein G (Invitrogen) mix on rotator at 4 °C overnight. After washing, reversal of crosslink and DNA purification, equal amounts of IP (by NFKB1 antibody or IgG control) and input DNA was used as a template for conventional PCR assay using specific primers targeting a region within 100 bp of the putative binding site.

### Rescue experiments

miR-508-3p precursor together with the negative control were transfected in MKN28 and SGC-7901 cells. And 24 h after precursor transfection, NFKB1-expression plasmid and empty plasmid (pcDNA3, Life Technologies, Grand Island, NY) were subsequently transfected with FuGENE HD Transfection Reagent (Roche, Nutley, NJ). After another 24 h, cells were collected for functional study (MTT proliferation assays and monolayer colony formation assays).

### Statistical analysis

The Student *T* test was used to compare the differences in biological behavior between siNFKB1, siRELA and siScramble control transfected cells. It is also used to compare the functional differences between miR-508-3p transfected cells and scramble miRNA transfectant counterparts. Expression of NFKB1, RELA or miR-508-3p in GC cell lines, primary cancerous tissues and the corresponding paired noncancerous tissues were compared by Mann–Whitney *U* test and paired *T* test. Correlations between NFKB1 and RELA expression and clinicopathologic parameters were assessed by Pearson correlation analysis. The Kaplan-Meier method was employed to estimate the survival rates for each variable. The equivalences of the survival curves were tested by log-rank statistics. All statistical analysis was performed by SPSS software (Version 16.0; SPSS Inc). A two-tailed *P*-value of less than 0.05 was considered statistically significant.

## Additional file

Additional file 1: Figure S1. Functional study of NFKB1 and RELA knockdown. (A) The representative figures of cell invasion in three GC cell lines (MKN28, MGC-803, and SGC-7901) after treatment with siScramble, siNFKB1 or siRELA. (B) The cell migration images of GC cell upon NFKB1 or RELA knockdown. (C) The representative cell cycle distribution images from FACS flow cytometry analysis in siScramble, siNFKb1 or siRELA transfectants. (D) siNFKB1 and siRELA significantly supressed the xenograft formation in a 28-day inoculation using MGC-803 cells. (TIF 5626 kb)

## Abbreviations

ChIP: chromatin immunoprecipitation
ESCC: esophageal squamous cell carcinoma
GC: gastric cancer
INJJ5J: inositol polyphosphate-5-phosphatase J
INPP4A: inositol polyphosphate 4-phosphatase type I
miR-508-3p: microRNA-508-3p
miRNA: microRNA
NF-κB: nuclear factor-kappa B
PTEN: phosphatase and tensin homologue
qRT-PCR: quantitative real-time polymerase chain reaction
RCC: renal cell carcinoma
UTR: untranslated region
