# Supplementary figures and images for: miR-508-3p concordantly silences NFKB1 and RELA to inactivate canonical NF-κB signaling in gastric carcinogenesis
Source: Mol Cancer. 2016 Jan 22;15:9. doi: 10.1186/s12943-016-0493-7 (PMC4724081; doi:10.1186/s12943-016-0493-7)

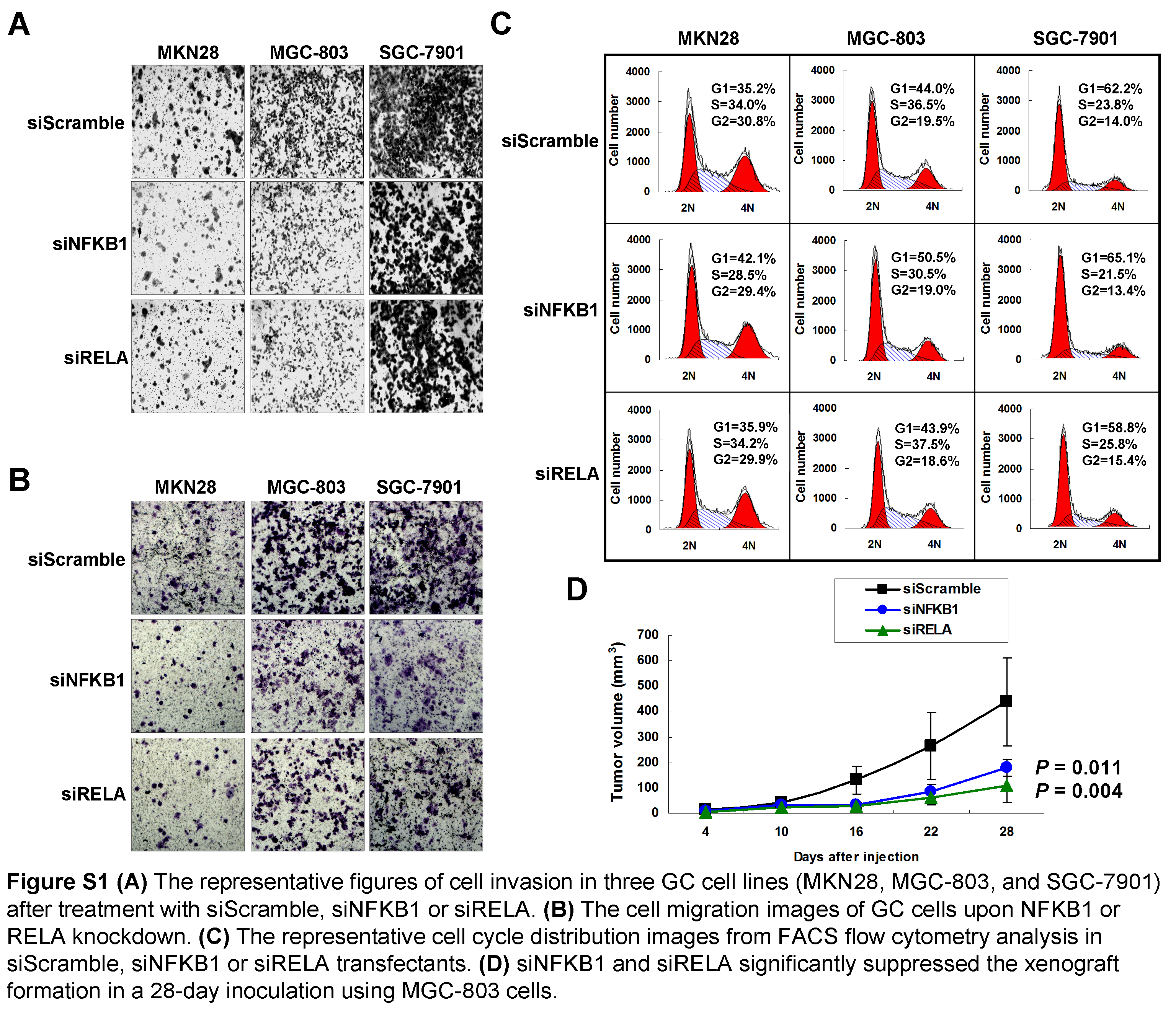

Supplement: Additional file 1: Figure S1. — Functional study of NFKB1 and RELA knockdown. (A) The representative figures of cell invasion in three GC cell lines (MKN28, MGC-803, and SGC-7901) after treatment with siScramble, siNFKB1 or siRELA. (B) The cell migration images of GC cell upon NFKB1 or RELA knockdown. (C) The representative cell cycle distribution images from FACS flow cytometry analysis in siScramble, siNFKb1 or siRELA transfectants. (D) siNFKB1 and siRELA significantly supressed the xenograft formation in a 28-day inoculation using MGC-803 cells. (TIF 5626 kb) [file 12943_2016_493_MOESM1_ESM.tif]
